# Integrated Analysis of mRNA and lncRNA Expression Profiles Reveals Regulatory Networks Associated with Decompensated Cirrhosis

**DOI:** 10.1155/2022/1805216

**Published:** 2022-11-16

**Authors:** Li Zhang, Xiaoyu Fang, Suhua Wang, Shasha Ma, Jinyan Zhang, Xiang Dong, Jing Dai, Chuanmiao Liu, Yu Gao

**Affiliations:** ^1^Department of Infectious Diseases, The First Affiliated Hospital of Bengbu Medical College, Bengbu Medical College, Bengbu 233030, China; ^2^Clinical Laboratory, The First Affiliated Hospital of Bengbu Medical College, Bengbu Medical College, Bengbu 233030, China; ^3^School of Life Science, Bengbu Medical College, Bengbu 233030, China; ^4^Bengbu Medical College Key Laboratory of Cancer Research and Clinical Laboratory Diagnosis, Bengbu Medical College, Bengbu 233030, China; ^5^Anhui Province Key Laboratory of Translational Cancer Research, Bengbu Medical College, Bengbu 233030, China

## Abstract

The stage of decompensation is termed end-stage liver cirrhosis. Patients with decompensated cirrhosis (DCC) often have a variety of comorbidities that contribute to exacerbation of the disease and its high mortality rate. By comparing differential gene expression, transcriptomic analysis is useful for exploring relevant functional changes during disease progression. The purpose of this study was to identify differentially expressed long noncoding RNAs (lncRNAs) and mRNAs in patients with decompensated cirrhosis and to further explore the functions as well as interactions between lncRNAs and mRNAs. Four patients with decompensated cirrhosis and four controls with liver cirrhosis were recruited in this study. RNA was isolated from peripheral blood mononuclear cells, and RNA-seq was used for transcriptome analysis. The functions of differentially expressed mRNAs were revealed by Gene Ontology (GO) functional annotation and Kyoto Encyclopedia of Genes and Genomes (KEGG) pathway enrichment analyses, and a regulatory network was also constructed. A total of 1046 differentially expressed mRNAs and 1175 lncRNAs were identified between the decompensated cirrhosis patients and cirrhosis controls. Functional enrichment analyses indicated enrichment of genes involved in pathways related to inflammation and cellular metabolic activities. In addition, the findings suggested that the phagosome/endosome/autophagy-lysosome pathway might play an important role in cirrhotic decompensation. In summary, this study identified differentially expressed mRNAs (DE-mRNAs) and DE-lncRNAs and predicted the biological processes and signaling pathways involved in cirrhotic decompensation, which might provide new ideas for further revealing the molecular mechanism of DCC pathogenesis.

## 1. Introduction

Liver cirrhosis (LC) is a clinical chronic progressive liver disease caused by one or more pathogenic factors. Although liver function can be compensated for at the early stage, when cirrhosis develops to a degree beyond the compensatory capacity, it is known as decompensated cirrhosis (DCC), with main clinical manifestations of liver dysfunction and portal hypertension [1]. Related to the pathogenesis of liver cirrhosis, the stage of decompensation is termed end-stage liver cirrhosis. To date, there is no particularly effective treatment for the late stage of liver cirrhosis. Indeed, it can only be treated according to complications; however, if the etiology of liver cirrhosis can be identified, it can be actively treated according to this etiology, which may further delay progression of liver cirrhosis [2]. Thus, it is imperative to identify the molecular mechanism of liver cirrhosis in the decompensated period to facilitate the development of novel therapeutic targets for treatment of decompensated cirrhosis.

The decompensated stage of cirrhosis is a malignant disease triggered by degenerative necrosis of hepatocytes, regeneration, and structural damage to liver lobules and pseudobullet formation. With advances in the study of hepatocyte immunity, the process of cirrhosis failure is considered to be an immune system-mediated inflammatory response and imbalance in liver tissue repair [3]. During this process, hepatic stellate cells interact with multiple immune cells to form a complex immune regulatory network that influences liver fibrosis and the disease process of cirrhosis [4].

It is well known that in the disease state, expression levels of many genes can be significantly altered, leading to an abnormal transcriptome. Therefore, screening and identifying abnormally expressed RNAs in the transcriptome is an effective way to elucidate the molecular mechanism of disease. Moreover, interaction between different types of RNA molecules is crucial for the stability, maintenance, and regulation of the transcriptome. Based on the overall level of RNA in cells or tissues, RNA-seq (RNA sequencing) technology is used to study the regulation of gene expression and function to reveal specific biological processes. RNA-seq is also an efficient method to explore the molecular mechanisms involved in disease development. RNA-seq technology has been widely applied for analyzing the pathogenesis of various liver diseases and for screening relevant markers [5]. Furthermore, it has been employed to assess changes in gene expression profiles and related regulatory networks in liver tissues of patients with cirrhosis related to various etiologies, such as hepatitis B virus [6], hepatitis C virus [7], alcoholic liver disease [8], and nonalcoholic fatty liver disease [9]. Results to date suggest that transcriptomic studies based on RNA-seq might be an important tool in exploring the mechanisms of liver steatosis, fibrosis, and cirrhosis. Nevertheless, there is no report about the role of the RNA expression profile or RNA regulatory relationship in decompensated cirrhosis.

In this study, total RNAs extracted from whole blood of patients with decompensated cirrhosis (DCC) and individuals with liver cirrhosis (LC) were sequenced. Profiles of mRNAs and lncRNAs (long noncoding RNAs) were analyzed to identify differentially expressed mRNAs and lncRNAs associated with decompensated cirrhosis. lncRNA target genes were predicted based on the theory of cis-acting regulatory elements, and pathophysiological processes were analyzed by Gene Ontology (GO) functional annotation and Kyoto Encyclopedia of Genes and Genomes (KEGG) pathway enrichment. A regulatory network with hub genes was evaluated. The aim of this study was to integrate the analysis of mRNAs and lncRNAs to reveal regulatory networks associated with DCC, which might enhance our understanding of the molecular mechanisms underlying the development of DCC.

## 2. Materials and Methods

### 2.1. Decompensated Cirrhosis Patients and Liver Cirrhosis Controls

Four decompensated cirrhosis (DCC) patients (two males and two females) and four liver cirrhosis (LC) patients (two males and two females) were recruited for this study from the First Affiliated Hospital of Bengbu Medical College, Anhui, China. The diagnosis of DCC was based on “EASL Clinical Practice Guidelines for the management of patients with decompensated cirrhosis” [10]. This study was conducted according to the guidelines of the Declaration of Helsinki and approved by the institutional ethics board of Bengbu Medical College (No. 2020LK078), and informed consent was obtained from all participants.

### 2.2. RNA Sample Qualification and Quantification

From each participant, 2.5 mL of blood was collected into a PAXgene Blood RNA Tube (PreAnalytiX, Qiagen/BD, USA) according to the manufacturer's protocol. After blood collection, the PAXgene Blood RNA Tubes were gently inverted 10 times and were incubated for 2 h at room temperature. Then, the tubes were stored at -20°C for 24 hours followed by freezing at -80°C until RNA extraction. Total RNA was isolated from blood samples collected in PAXgene Blood RNA Tubes using a PAXgene Blood RNA Kit IVD following the manufacturer's guideline (PreAnalytiX, Qiagen/BD, USA). The RNA samples were assessed for possible contamination and degradation using 1% agarose gel electrophoresis. RNA purity was examined using a NanoPhotometer^®^ spectrophotometer (Implen, CA, USA), and RNA concentration was measured using a Qubit^®^ RNA Assay Kit and a Qubit^®^ 2.0 Fluorometer (Life Technologies, CA, USA). Finally, the integrity of the RNA was assessed using RNA Nano 6000 Assay Kit of the Bioanalyzer 2100 system (Agilent Technologies, CA, USA).

### 2.3. lncRNA and mRNA Library Construction and Sequencing

The lncRNA and mRNA library was constructed by using NEBNext^®^ Ultra^™^ Directional RNA Library Prep Kit for Illumina^®^ (NEB, USA). The insert size of the acquired library was checked with an Agilent 2100 Bioanalyzer. After library preparation, the samples were sequenced using the Illumina HiSeq 2500 platform. Paired-end clean reads were aligned to the human reference genome using the program HISAT2 (hierarchical indexing for spliced alignment of transcripts 2) [11].

### 2.4. Identification of Differentially Expressed mRNAs (DE-mRNAs) and DE-lncRNAs

All transcripts for lncRNAs and mRNAs were merged using the Cuffmerge tool, which was one part of Cufflinks [12]. Novel lncRNAs were named following the rules of HGNC (HUGO Gene Nomenclature Committee). Quantification of the transcripts and genes was performed using StringTie software, and the reads per kilobase of transcript per million reads mapped (RPKM) was obtained. Differentially expressed mRNAs (DE-mRNAs) and lncRNAs (DE-lncRNAs) between the DCC patients and LC controls were identified using the edgeR algorithm in the R package [13]. The resulting *P* values were adjusted using Benjamini and Hochberg's approach for controlling the false discovery rate. The thresholds for screening DE-mRNAs and DE-lncRNAs were ∣log2 (fold change) | >1 and *P*.adj < 0.05.

### 2.5. Target Gene Prediction of lncRNAs

Target genes of lncRNAs were predicted by using cis-acting target gene prediction. Based on the theory of cis-acting regulatory elements, the protein-coding genes located within 10/100 kb upstream and downstream of lncRNAs were selected as potential cis-acting targets [14]. In this study, the threshold of colocalization was set to 100 kb upstream and downstream of lncRNAs.

### 2.6. Alternative Splicing Event Analysis

Five types of alternative splicing events (ASEs) were identified based on splice junction reads, including skipped exons (SEs), retained introns (RIs), mutually exclusive exons (MXEs), alternative 5′ splice sites (A5SSs), and alternative 3′ splice sites (A3SSs). Regulated alternative splicing events (RASEs) between DCC patients and LC controls were defined and quantified using rMATS software [15]. The *P* value was calculated by the likelihood-ratio test to represent the difference between the two groups at IncLevel (inclusion level), and the FDR value was obtained by using the Benjamini and Hochberg algorithm.

### 2.7. Functional Enrichment Analysis

GO and KEGG functional pathway analyses were carried out to predict the function of differentially expressed genes, lncRNA target genes, and genes related to regulated ASEs. Enrichment was considered to be significant when the corrected *P* values were less than 0.05.

### 2.8. Protein-Protein Interaction Enrichment Analysis

Protein-protein interaction (PPI) enrichment analysis was performed by using the Metascape online analysis tool according to the STRING and BioGRID databases [16]. The molecular complex detection (MCODE) algorithm [17] was applied to identify densely connected network components in the PPI network and to annotate the biological functions of each cluster, as well as to identify key genes that influence the process of DCC.

### 2.9. qRT-PCR Validation of Selected Differentially Expressed RNAs

To confirm the validity of the RNA-seq results, qRT-PCR was performed for some randomly selected differentially expressed RNAs identified by RNA-seq. The sequences of primers used for differentially expressed RNA validation are presented in Table S1 in the supplemental materials. GAPDH (glyceraldehyde-3-phosphate dehydrogenase) was used as the internal reference gene.

### 2.10. Statistical Analysis

All the above analyses were carried out by using R software version 4.0.3. Differences between the two groups were analyzed using independent samples *t* tests and paired *t* tests. Correlation analysis was based on Pearson's correlation test. Statistical significance was defined as *P* < 0.05 (^∗^), *P* < 0.01 (^∗∗^), and *P* < 0.001 (^∗∗∗^).

## 3. Results

### 3.1. Differentially Expressed mRNAs and lncRNAs in PBMCs of Decompensated Cirrhosis Patients and Liver Cirrhosis Controls

**Figure 1:**
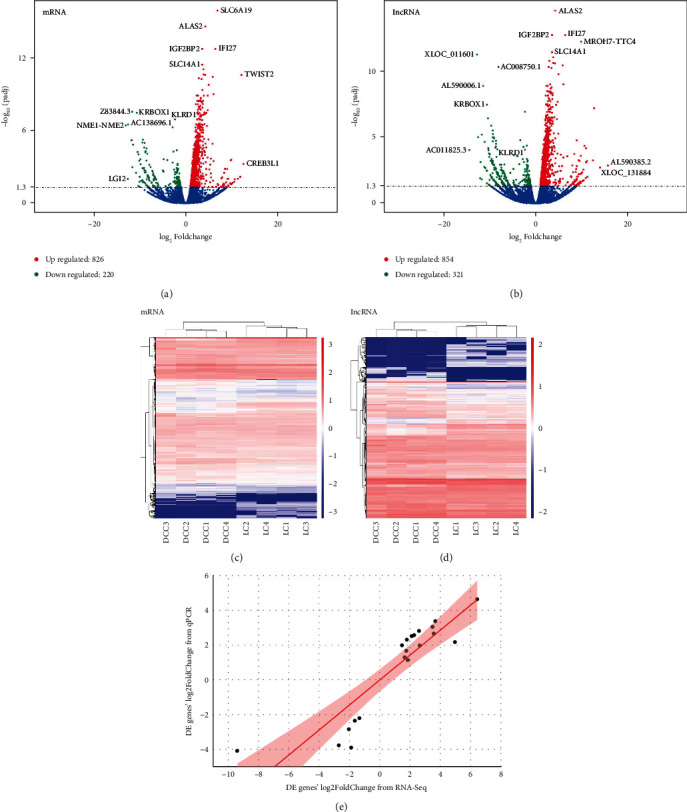
Overview of differentially expressed mRNAs and lncRNAs in PBMCs of decompensated cirrhosis patients (DCC) and liver cirrhosis controls (LC). (a) Differentially expressed mRNAs between the DCC group and the LC group. (b) Differentially expressed lncRNAs between the DCC group and the LC group. (c) Two-way clustering heatmap of the differentially expressed mRNAs. (d) Two-way clustering heatmap of the differentially expressed lncRNAs. (e) Correlation of log2 fold change between RNA-seq and RT-qPCR for significantly differentially expressed genes.

Four DCC patients and four LC controls were included in this study. There was no genetic relation between all patients in this study. The average age of DCC patients was 47.5 (range, 31-68) years, and the average age of LC control was 49.3 (range, 39-63). None of the participants had diabetes, hypertension, cancer, cardiovascular disease, immune-related diseases, or smoking history. Total RNA was extracted from the blood of each participant and sequenced. The sequence data of mRNAs and lncRNAs have been submitted to the NCBI SRA database under accession number PRJNA798844. The RNA expression levels in the RNA-seq results were expressed as FPKM (fragments per kilobase of transcript sequence per millions base pairs sequenced). Among 16697 detected mRNAs, 1046 differentially expressed mRNAs were identified in DCC samples compared to LC samples, including 826 significantly upregulated mRNAs and 220 significantly downregulated mRNAs (Figure 1(a) and Table S2 in supplemental materials). Among all 23083 detected lncRNAs, 1175 lncRNAs were found to be significantly aberrantly expressed, including 854 upregulated and 321 downregulated lncRNAs in DCC patients compared to LC patients (Figure 1(b) and Table S3). Two-way clustering heatmaps of the differentially expressed mRNAs and lncRNAs at the transcript levels are shown in Figures 1(c) and 1(d), with DCC samples being significantly separated from LD samples, indicating that the results of the differential expression analysis are reliable. To verify the differentially expressed RNAs obtained from RNA-seq, the RNA expression levels of 20 genes were also assessed by qRT-PCR. Between the two techniques, Pearson's correlation coefficient of fold change in gene expression levels was 0.8939 (*P* < 0.001) (Figure 1(e)).

### 3.2. Functional Enrichment Analysis of Differentially Expressed mRNAs

**Figure 2:**
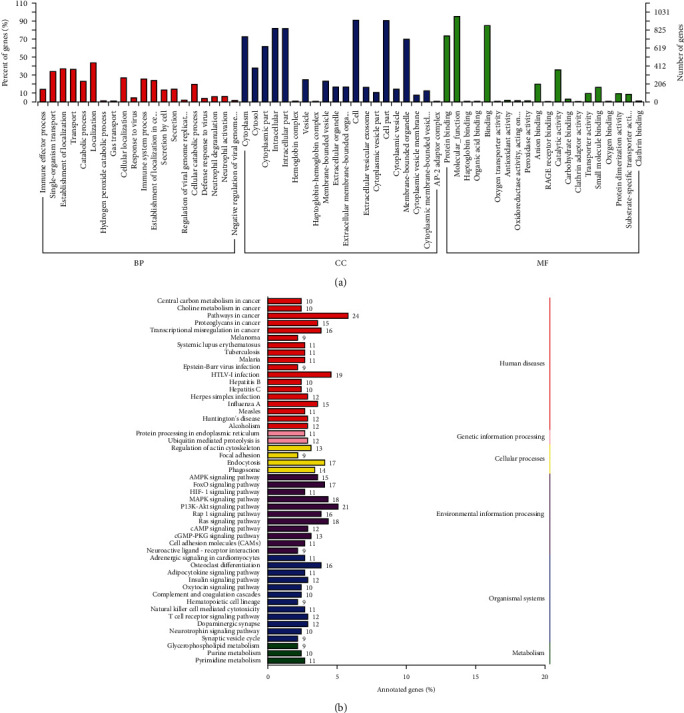
The potential biological functions of differentially expressed mRNAs between DCC and LC. (a) The top twenty Gene Ontology (GO) terms belonging to the categories biological process (BP), cellular component (CC), and molecular function (MF) of DE-mRNAs. (b) Kyoto Encyclopedia of Genes and Genomes (KEGG) pathway enrichment.

To explore the potential pathogenic mechanism of DCC, Gene Ontology (GO) enrichment and Kyoto Encyclopedia of Genes and Genomes (KEGG) pathway analyses were performed on the significantly differentially expressed mRNAs. The top twenty GO terms belonging to the categories biological process (BP), cellular component (CC), and molecular function (MF) are provided in Figure 2(a). In total, 231 terms were significantly enriched in the biological processes category (Table S4), including immune effector process (GO:0002252), hydrogen peroxide catabolic process (GO:0042744), response to virus (GO:0009615), cellular localization (GO:0051641), and gas transport (GO:0015669). The plot of lineage DAG (directed acyclic graph) on biological process (BP) was illustrated in Figure S1(a) in the supplemental materials. There were 60 markedly enriched GO terms belonging to the cellular component category (Table S4), including extracellular organelle (GO:0043230), membrane-bounded vesicle (GO:0031988), hemoglobin complex (GO:0005833), and haptoglobin-hemoglobin complex (GO:0031838). The DAG plot for cellular component (CC) is shown in Figure S1(b) in the supplemental materials. For the molecular function category, 45 GO terms were notably enriched (Table S4), including oxygen transporter activity (GO:0005344), haptoglobin binding (GO:0031720), organic acid binding (GO:0043177), anion binding (GO:0043168), and peroxidase activity (GO:0004601); the DAG plot is given in Figure S1(c) in the supplemental materials. Moreover, the results of KEGG pathway enrichment analysis showed 18 pathways to be significantly enriched at a *P* value less than 0.05, including malaria (ko05144), nitrogen metabolism (ko00910), adipocytokine signaling pathway (ko04920), butirosin and neomycin biosynthesis (ko00524), FoxO signaling pathway (ko04068), osteoclast differentiation (ko04380), central carbon metabolism in cancer (ko05230), and AMPK signaling pathway (ko04152) (Table S5). To display the results of KEGG analysis more clearly, the enriched pathways were grouped into the categories of human diseases, genetic information processing, cellular processes, environmental information processing, organismal systems, and metabolism (Figure 2(b)).

### 3.3. Functional Enrichment Analysis of DE-lncRNA Target Genes

**Figure 3:**
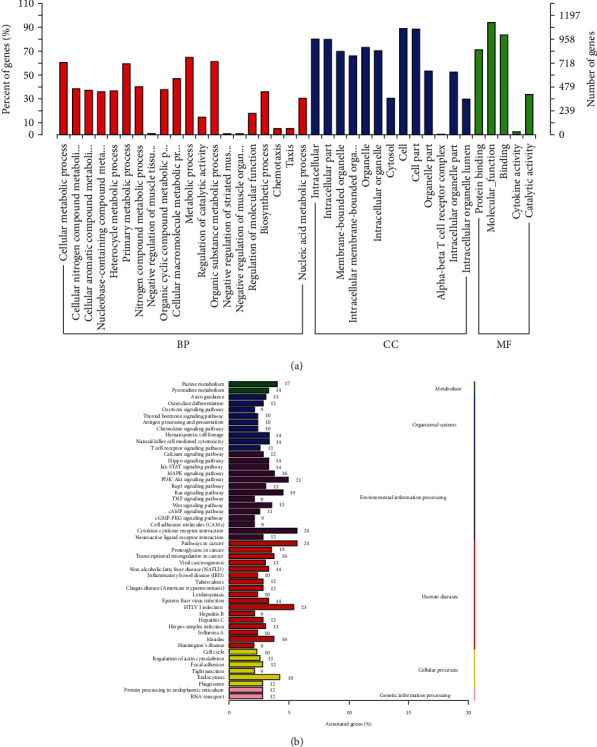
The potential biological functions of DE-lncRNA target genes. (a) Gene Ontology (GO) functional enrichment of DE-lncRNA target genes. (b) KEGG pathway enrichment of DE-lncRNA target genes.

Based on the theory of cis-acting regulatory elements, the protein-coding genes located within 100 kb of lncRNAs were selected as potential cis-acting targets. Based on 1177 DE-lncRNAs, 1231 cis-acting target mRNAs were identified (Table S6). There were 34 markedly enriched GO terms belonging to the BP category, 13 belonging to the CC category, and 5 belonging to the MF category (Figure 3(a) and Table S7). According to the results of GO enrichment analysis, the DE-lncRNA target genes are mainly enriched in cellular metabolic processes, including cellular nitrogen compound metabolic processes, cellular aromatic compound metabolic processes, nucleobase-containing compound metabolic processes, heterocycle metabolic processes, primary metabolic processes, and nitrogen compound metabolic processes. The results of KEGG pathway enrichment analysis revealed that 16 pathways are significantly enriched at a *P* value less than 0.05, including hematopoietic cell lineage (ko04640), graft-versus-host disease (ko05332), and pyrimidine metabolism (ko00240) (Figure 3(b)). The full list of the KEGG analysis results is shown in Table S8 in the supplemental materials.

### 3.4. Analysis of Regulated Alternative Splicing Events in PBMCs of Decompensated Cirrhosis Patients and Liver Cirrhosis Controls

**Figure 4:**
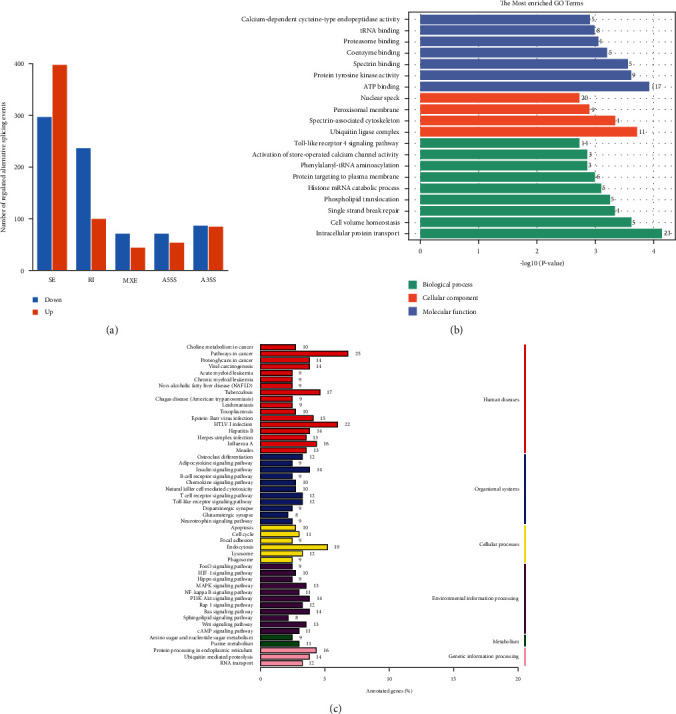
Enrichment biological function of regulated alternative splicing events in PBMCs between DCC and LC. (a) The number of significant regulated alternative splicing events, including events of skipped exon (SE), retained intron (RI), mutually exclusive exons (MXE), 5′ splice site (A5SS), and 3′ splice site (A3SS) events. (b) GO functional enrichment of different RASEs. (c) KEGG pathway enrichment analysis results.

To examine regulated ASEs in DCC patients, RASEs (regulated alternative splicing events) were analyzed using transcriptional sequencing data obtained from PBMCs of the DCC cirrhosis patients and LC controls. A total of 1446 RASEs were significant between the two groups. Of the RASE types, there were 696 SE events, 338 RI events, 114 MXE events, 126 A5SS events, and 172 A3SS events detected (Figure 4(a)). The complete list of RASEs is shown in Supplementary Table S9. GO enrichment results revealed RASEs in PBMCs of DCC patients to be mainly enriched in the ubiquitin ligase complex, ATP binding, intracellular protein transport protein, tyrosine kinase activity, spectrin-associated cytoskeleton, spectrin-binding coenzyme, binding peroxisomal membrane, and cell volume homeostasis, among others (Figure 4(b)). Results of KEGG pathway enrichment showed the RASEs to be involved in pathways associated with cellular processes, metabolism, environmental information processing, organismal systems, and so on (Figure 4(c)).

### 3.5. Protein-Protein Interaction Analysis Associated with Decompensated Cirrhosis

**Figure 5:**
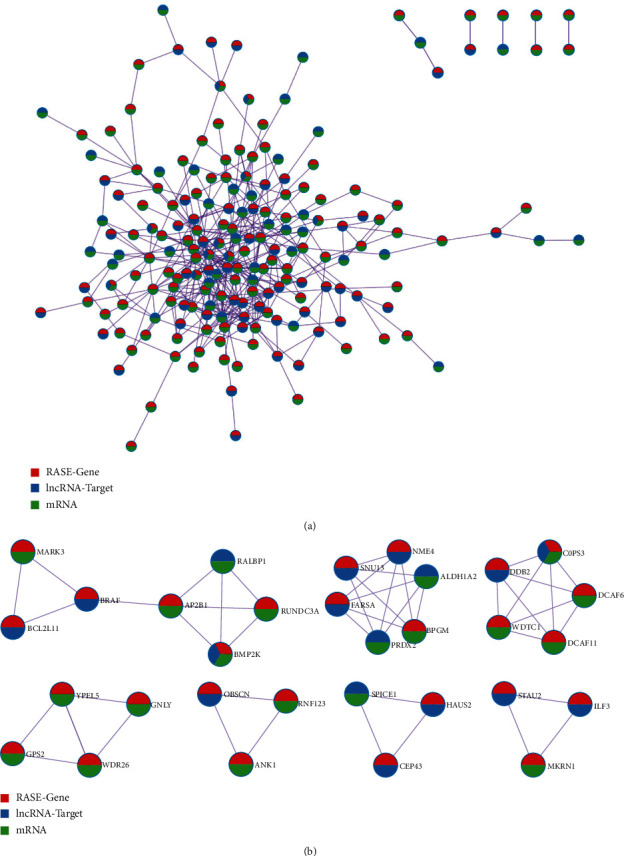
Protein-protein interaction analysis associated with decompensated cirrhosis. (a) PPI (protein-protein interaction) network obtained from DE-mRNAs, DE-lncRNA target genes, and RASE genes. (b) MCODE algorithm was then applied to this network to identify neighborhoods where proteins were densely connected. Network nodes were displayed as pies. Color code for pie sector represented the gene list with the different color.

To identify genes that play a major role in the development of cirrhosis, a protein interaction network analysis was performed between mRNAs, lncRNA target genes, and RASE-related genes. The PPI (protein-protein interaction) network obtained from the analysis consisted of 193 nodes and 390 edges (Figure 5(a)). Further enrichment analysis showed that it is mainly focused on biological processes such as proteolysis involved in cellular protein catabolic processes (log10(*P*) = −10.7), cellular protein catabolic processes (log10(*P*) = −10.4), and ubiquitin-dependent protein catabolic processes (log10(*P*) = −10.1). Based on the PPI network results, the molecular complex detection (MCODE) algorithm was applied to model the interaction network; densely linked protein clusters in the network were detected, and the biological functions of each cluster were annotated. A total of seven clusters were analyzed, consisting of 31 nodes and 46 edges (Figure 5(b)), containing the key genes that influence the development of cirrhosis. Each cluster contained one “seed” gene, and the “seed” genes of the seven MCODE clusters were RUNDC3A (RUN domain containing 3A), BPGM (bisphosphoglycerate mutase), DCAF11 (DDB1- and CUL4-associated factor 11), GPS2 (G protein pathway suppressor 2), OBSCN (obscurin, cytoskeletal calmodulin, and titin-interacting), CEP43 (centrosomal protein 43), and STAU2 (Staufen double-stranded RNA binding protein 2).

### 3.6. Overview of Phagosome/Endosome/Autophagy-Lysosome Pathways in PBMCs from Patients with Decompensated Cirrhosis

**Figure 6:**
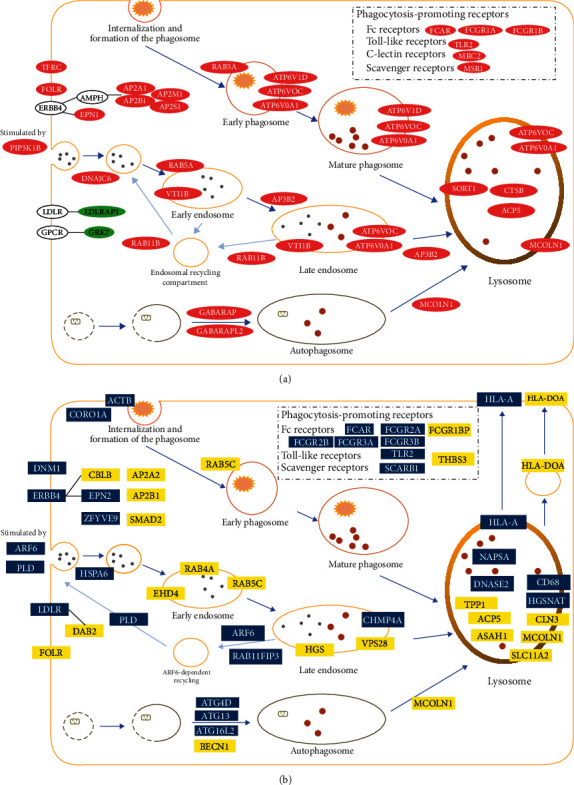
Overview of phagosome/endosome/autophagy-lysosome pathways in PBMCs from patients with decompensated cirrhosis. (a) The overview of phagosome/endosome/autophagy-lysosome pathways based on the expression levels of differentially expressed mRNAs. The figure was drawn according to the information from KEGG PATHWAY database. The molecules in red were upregulated in PBMCs from DCC patients, and the molecules in green were downregulated in PBMCs from DCC patients. (b) The overview of phagosome/endosome/autophagy-lysosome pathways according to the target genes of DE-lncRNAs and genes involved in RASEs. The molecules in blue were lncRNA target genes, and the molecules in yellow were genes involved in RASEs.

Based on the expression levels of differentially expressed mRNAs and the results of functional enrichment, an overview of phagosome/endosome/autophagy-lysosome pathway in DCC is depicted in Figure 6(a). Overall, 29 upregulated mRNAs and 2 downregulated mRNAs were identified in PBMCs of the DCC patients compared to the LC controls. From the early phagosome to the mature phagosome, expression levels of ATP6V0A1 (ATPase H+ transporting V0 subunit A1), ATP6V0C (ATPase H+ transporting V0 subunit C), and ATP6V1D (ATPase H+ transporting V1 subunit D), which encode ATPase, increased in PBMCs from patients with decompensated cirrhosis. Phagocytosis-promoting receptors were also upregulated, including Fc receptors (FCAR (Fc alpha receptor), FCGR1A (Fc gamma receptor Ia), and FCGR1B (Fc gamma receptor Ib)), Toll-like receptor (TLR2 (Toll-like receptor 2)), C-lectin receptor (MRC2 (mannose receptor C type 2)), and scavenger receptor (MSR1 (macrophage scavenger receptor 1)). In the endosome-lysosome pathway, clathrin-dependent endocytosis might be stimulated by overexpression of PIP5K1B (phosphatidylinositol-4-phosphate 5-kinase type 1 beta); increased expression of genes encoding ATPase was also observed in the late endosome. Additionally, expression of RAB11B (Ras-related protein Rab-11B) might play a role during endosomal recycling. The proteins encoded by GABARAP (GABA type A receptor-associated protein) and GABARAPL2 (GABA type A receptor-associated protein-like 2) might regulate the autophagy process, and those encoding acid hydrolases, sulfatases, phosphatases, and lysosomal membrane proteins were upregulated in DCC. According to the target genes of DE-lncRNAs and genes involved in RASEs, the overview of phagosome/endosome/autophagy-lysosome pathways with decompensated cirrhosis is displayed in Figure 6(b), including 28 DE-lncRNA target genes in the lysosome-related pathways. Among them, 5 genes are Fc receptors, which are types of phagocytosis-promoting receptors. In the endosome-lysosome pathway, ARF6 (ADP ribosylation factor 6) might play a role in the processes of clathrin-dependent endocytosis and ARF6-dependent endosome recycling. A total of 22 genes related to RASEs were identified in lysosome-related pathways. The genes EHD4 (EH domain containing 4), RAB4A (Ras-related protein Rab-4A), RAB5C (Ras-related protein Rab-5C), HGS (hepatocyte growth factor-regulated tyrosine kinase substrate), and VPS28 (ESCRT-I complex subunit VPS28) might perform certain functions during the formation of early to late endosomes. Genes encoding acid hydrolases and membrane proteins in lysosomes were also detected in DCC.

## 4. Discussion

Cirrhosis is fibrous and necrotizing inflammation of the liver and is an irreversible, progressive, and chronic liver disease caused by repeated stimulation of one or more mechanisms that cause liver injury. As the disease progresses, it gradually exceeds the ability of the liver to compensate for its own functions and progresses to the decompensated stage, the late stage of liver cirrhosis. At this time, DCC patients might have repeated ascites, a large spleen, esophageal variceal bleeding, hepatic encephalopathy, and other manifestations of liver function injury. Furthermore, there is a certain risk of carcinogenesis and even death due to acute complications and critical disease. However, there is still a lack of ideal and effective treatments for DCC. Studying lncRNA-mRNA regulatory networks in cirrhosis would improve the diagnosis and treatment of cirrhosis, even DCC, and reveal the initial disorder of disease and its potential mechanism. In this study, differentially expressed mRNAs and lncRNAs were obtained based on high-throughput RNA sequencing technology. Functional enrichment analyses of these differentially expressed mRNAs, lncRNA target genes, and RASEs were performed, and the results suggest that altered expression of genes involved in the phagosome/endosome/autophagy-lysosome pathways might be related to DCC.

The liver is an important immune organ of the body. According to advances in the study of hepatocyte immunity, DCC is considered to be a process of immune system-mediated inflammatory response and imbalance in liver tissue repair [18]. During this process, hepatic cells interact with multiple immune cells, forming a complex immune regulatory network to influence liver fibrosis and the cirrhosis disease process. Hence, the inflammatory response plays an important role in the development of cirrhosis and is important for immune regulation. Neutrophils and lymphocytes are the main cells of the immune system. The former might be chemotactic, phagocytic, and bactericidal, reflecting the ability of the body to respond to inflammation [19]; the latter could reflect the immune level and nutritional status of the body [20]. In general, patients with cirrhosis have immune dysfunction and abnormal secretion of various cytokines. A previous study reported significantly higher neutrophil and lymphocyte counts in patients who died due to cirrhosis decompensation than in the surviving group, suggesting that excess neutrophils and lymphocytes may be involved in progression of the disease in patients with cirrhosis decompensation [21]. In patients with DCC, there is often a multistimulated immune response, which stimulates overexpression of T lymphocytes and B lymphocytes and release of a large number of inflammatory mediators, such as interleukin-6 and interleukin-8, leading to an inflammatory response that drives release of neutrophils. Moreover, this process further exacerbates loss of the compensatory state [22]. The results of this study suggest that the complement and coagulation cascades, hematopoietic cell lineage, natural killer cell mediated cytotoxicity, antigen processing and presentation pathway, T cell receptor signaling pathway, and B cell receptor signaling pathway were involved in the regulation of cirrhosis decompensation.

Patients in the decompensated phase often experience a combination of infections of different sites, and ectopic intestinal flora and dysbiosis can lead to systemic inflammation [23]. Studies have shown that levels of inflammatory factors such as leukocytes, C-reactive protein, and interleukins differ in patients with different disease states in the decompensated phase and are significantly higher than in those in the compensated phase [24–26]. In addition, systemic inflammation exacerbates hemodynamic disturbances in patients with multiorgan and systemic dysfunction. In this stage, complex immune dysfunction, including innate and acquired immunity, often leads to an increased risk of bacterial infections in patients with symptoms such as combined spontaneous bacterial peritonitis. Here, the results of potential biological functions of DE-mRNAs and lncRNAs suggested that the DCC patients might have a greater risk of tuberculosis, inflammatory bowel disease, and leishmaniasis and might be equally susceptible to infection of Epstein-Barr virus, HTLV-1 (human T cell leukemia virus-1), herpes simplex, influenza A, or measles. These diseases or infections would induce systemic inflammation, which is the result of sustained action of the circulating immune cells released by necrotic hepatocytes with molecules associated with injury [27].

Bacterial infection in DCC patients causes cytokine release, activating intrinsic immune cells in vital organs to release cytokines, proteases, and reactive oxygen species to defend against the bacteria, but an excessive inflammatory response causes tissue damage, i.e., immunopathological changes. Due to a series of processes, such as production of large amounts of inflammatory factors, the acute-phase response, and cell migration and proliferation, systemic inflammation requires large amount of nutrients and energy; however, such patients usually have reduced food intake, weakness, and poor nutrition [28, 29]. Nonimmune tissues adapt to this change by reducing energy requirements and mitochondrial oxidative phosphorylation [30]. Increased numbers of monocytes and macrophages expressing MER receptor tyrosine kinase (MERTK), a class of immune cells that suppress the body's innate immune response to pathogens, have been detected in patients with DCC and slow plus acute liver failure in numbers associated with advanced liver disease and intestinal injury [31]. Neutrophil migration from the circulation to the site of infection or injury is a critical immune response that requires destruction of endothelial cells (ECs) inside blood vessels, and unregulated neutrophil transendothelial migration (TEM) is pathogenic. Macroautophagy/classical autophagy is an evolutionarily conserved process for delivering cytoplasmic contents to lysosomes for degradation, and classic autophagy has emerged as a central regulator of innate immune function, including cytokine production, immune cell differentiation, and pathogen clearance. Despite sufficient evidence that immune cell autophagy-associated genes regulate inflammation, little is known about the role of EC autophagy in this regard. Nonetheless, autophagy is a homeostatic regulator of many EC functions, most notably EC survival and developmental angiogenesis [32]. Generally, in the initial stages of liver disease, autophagy can maintain the stability of the body environment through the removal of damage or the regulation of inflammatory signaling [33]. However, a variety of liver diseases have been found to correlate with autophagy disorders. With the disease progresses, excessive activation of autophagy might be detrimental and promote disease progression. In the presence of abnormalities in energy metabolism, such as with starvation, oxidative stress, and organelle accumulation, autophagy may be activated or inhibited, with certain negative effects on liver function [34]. For example, it has been found that in patients with slow plus acute liver failure, mitochondrial element 2 (mitofusin 2, MFN2) has a role in promoting mitochondrial outer membrane fusion and inner membrane docking, thereby increasing autophagy levels in hepatocytes and reducing apoptosis [35]. In advanced hepatocellular carcinoma (HCC), tumor cells recruit energy through autophagy, thereby enhancing their ability to survive in hypoxic and low-nutrient environments and promoting cancer progression [36]. In other words, autophagy could support the growth of hepatocellular carcinoma by promoting aerobic glycolysis in tumors [37]. In the present study, the results showed that the expression of mRNAs in the phagosome/endosome/autophagy-lysosome pathways in PBMCs from patients with decompensated cirrhosis was significantly upregulated compared to that in LC controls. It suggested that innate immunity in DCC patients might be overactivated, which could lead to the development of autoinflammatory responses and autoimmune diseases, and cause serious damage to the organism.

There are several limitations to this study. The significant limitation is the small number of decompensated cirrhosis (DCC) patients and liver cirrhosis (LC) controls. More samples are urgently needed to analyze the differentially expressed mRNAs and lncRNAs in the DCC and LC individuals and to investigate the role of phagosome/endosome/autophagy-lysosome pathway in cirrhotic decompensation. Also, it would be better to compare the profiles of mRNAs and lncRNAs to DCC and LC patients, which would prove more points about the process of liver fibrosis and the disease process of cirrhosis.

## 5. Conclusions

Through transcriptomic studies in patients with decompensated cirrhosis, differentially expressed mRNAs and lncRNAs associated with the pathophysiological process of decompensated cirrhosis were identified. It would provide possible new targets and pathways for subsequent research and antifibrotic therapy. At the same time, the analysis of alternative splicing events showed that variable splicing transcripts also played a relevant role in the pathogenesis of DCC, which might provide new ideas for further revealing the molecular mechanism of DCC pathogenesis.

## Data Availability

The authors confirm that the data supporting the findings of this study are available within the article and its supplementary materials. RNA-seq has been deposited in the Sequence Read Archive (SRA) database under PRJNA798844.

## Supplementary Materials

Supplementary 1 Table S1: primer sequences for qRT-PCR.

Supplementary 2 Table S2: the list of DE-mRNAs in PBMCs between DCC patients and LC controls.

Supplementary 3 Table S3: the list of DE-lncRNAs in PBMCs between DCC patients and LC controls.

Supplementary 4 Table S4: the result of GO functional enrichment analysis of differentially expressed mRNAs.

Supplementary 5 Table S5: the result of KEGG pathway analysis of differentially expressed mRNAs.

Supplementary 6 Table S6: the list of cis-acting target genes according DE-lncRNAs.

Supplementary 7 Table S7: the result of GO functional enrichment analysis of DE-lncRNA target genes.

Supplementary 8 Table S8: the result of KEGG pathway analysis of DE-lncRNA target genes.

Supplementary 9 Table S9: the list of regulated alternative splicing events decompensated cirrhosis.

Supplementary 10 Figure S1: the plots of lineage DAG (directed acyclic graph) on potential biological functions of differentially expressed mRNAs between DCC and LC. (a) The plot of lineage DAG (directed acyclic graph) on biological process (BP) category. (b) The plot of lineage DAG on cellular component (CC) category. (c) The plot of lineage DAG on molecular function (MF) category.
