# Supplementary material for: Integrated Analysis of mRNA and lncRNA Expression Profiles Reveals Regulatory Networks Associated with Decompensated Cirrhosis
Source: J Immunol Res. 2022 Nov 16;2022:1805216. doi: 10.1155/2022/1805216 (PMC9691389; doi:10.1155/2022/1805216)
Supplement: Supplementary 1 — Table S1: primer sequences for qRT-PCR. [file 1805216.f1.docx]

**Table S1.** Primer sequences for qRT-PCR

| **Gene Symbol** | **Forward primer sequence (5’-3’)** | **Reverse primer sequence (5’-3’)** |
| --- | --- | --- |
| ATG9A | CCAGAACTACATGGTGGCACT | GTCCCCAGAAGAGGATCAGC |
| BCAM | CAGGTCACAATGCACGACAC | CACCACGCACACGTAGTCT |
| CCL5 | CCAGCAGTCGTCTTTGTCAC | CTCTGGGTTGGCACACACTT |
| COL6A2 | GACTCCACCGAGATCGACCA | CTTGTAGCACTCTCCGTAGGC |
| FGFBP2 | CCTTACTGGAATCAAGCCCTG | ACACGGATGGCCTAAGCAC |
| GABARAP | AGAAGAGCATCCGTTCGAGA | CCAGGTCTCCTATCCGAGCTT |
| GABARAPL2 | ACTCGCTGGAACACAGATGC | TCTGAGAGCCTGAGACCTTTT |
| GADD45A | GAGAGCAGAAGACCGAAAGGA | CACAACACCACGTTATCGGG |
| GAPDH | ATGGGTGTGAACCATGAGAAGTA | GAGTGGGTGTCGCTGTTGAAGTC |
| GXYLT2 | GTGTCTCTATGTATTCCCCTGCC | TTATCGTCATGGTAGACGCCT |
| GZMB | CCCTGGGAAAACACTCACACA | GCACAACTCAATGGTACTGTCG |
| IFI27 | TGCTCTCACCTCATCAGCAGT | CACAACTCCTCCAATCACAACT |
| IFIT2 | AAGCACCTCAAAGGGCAAAAC | TCGGCCCATGTGATAGTAGAC |
| IFIT3 | TCAGAAGTCTAGTCACTTGGGG | ACACCTTCGCCCTTTCATTTC |
| MAP1LC3B | AAGGCGCTTACAGCTCAATG | CTGGGAGGCATAGACCATGT |
| MKRN1 | CATGGGGTTTGTAAGGAAGGAG | GCACACTACACTATACGGACTGT |
| MYBL2 | CCGGAGCAGAGGGATAGCA | CAGTGCGGTTAGGGAAGTGG |
| RORA | ACTCCTGTCCTCGTCAGAAGA | CATCCCTACGGCAAGGCATTT |
| SELENBP1 | TCATCTCCTCTCGCATCTATGTG | AAGGCCAGTTCGCACTTGG |
| SLC4A1 | CCTATACGCTTCCTCTTTGTGTT | CCATGTAGGCATCTATGCGGA |
| TMOD1 | GACCCTGATAATGCACTGCTG | ATCTTCTCGGTCCTTAAACTCCT |
