# Supplementary material for: Integrated Analysis of mRNA and lncRNA Expression Profiles Reveals Regulatory Networks Associated with Decompensated Cirrhosis
Source: J Immunol Res. 2022 Nov 16;2022:1805216. doi: 10.1155/2022/1805216 (PMC9691389; doi:10.1155/2022/1805216)
Supplement: Supplementary 10 — Figure S1: the plots of lineage DAG (directed acyclic graph) on potential biological functions of differentially expressed mRNAs between DCC and LC. (a) The plot of lineage DAG (directed acyclic graph) on biological process (BP) category. (b) The plot of lineage DAG on cellular component (CC) category. (c) The plot of lineage DAG on molecular function (MF) category. [file 1805216.f10.docx]

**Figure S1. The plots of lineage DAG (Directed Acyclic Graph) on potential biological functions of differentially expressed mRNAs between DCC and LC.**

(a) The plot of lineage DAG (Directed Acyclic Graph) on biological process (BP) category. (b) The plot of lineage DAG on cellular component (CC) category. (c) The plot of lineage DAG on molecular function (MF) category.


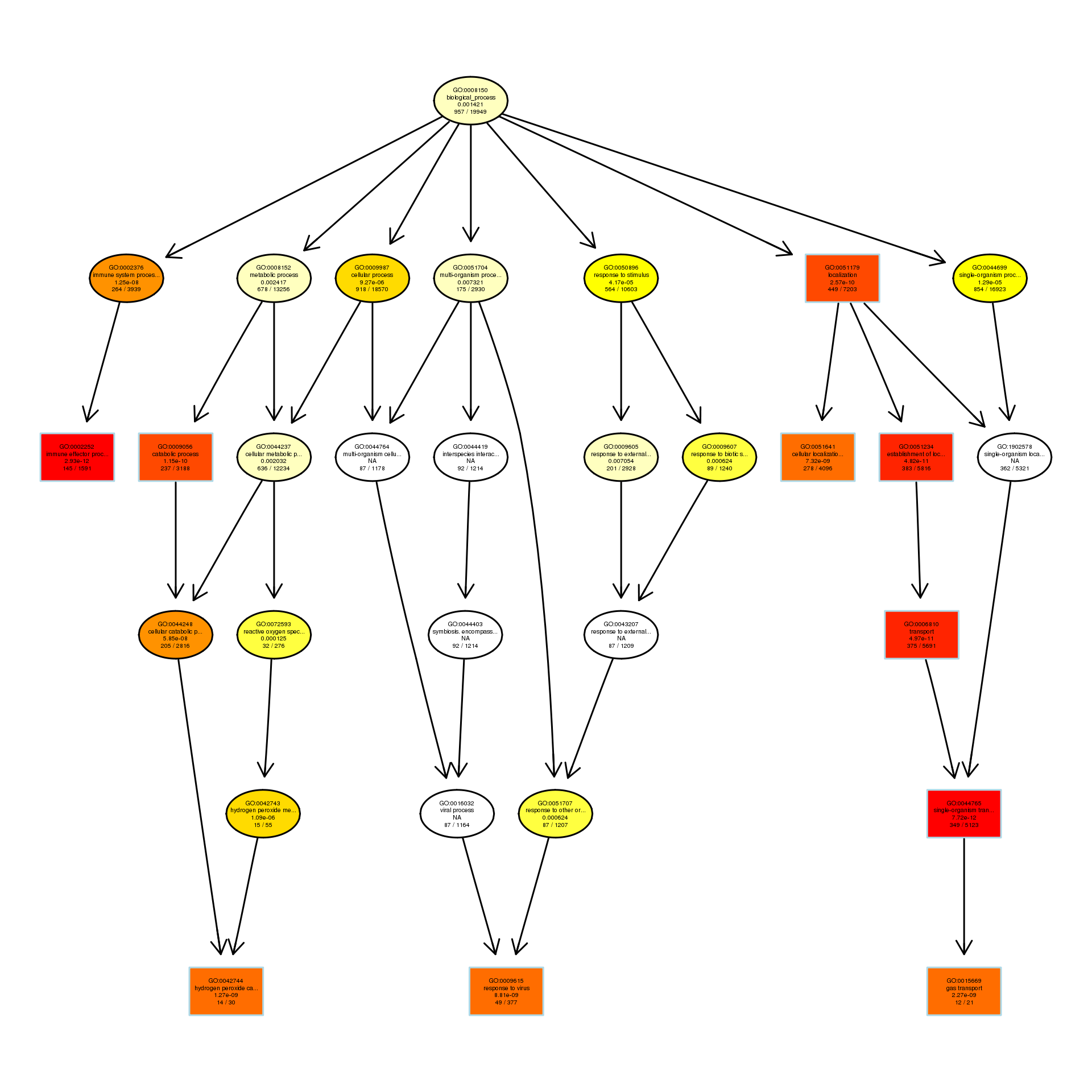


(a)


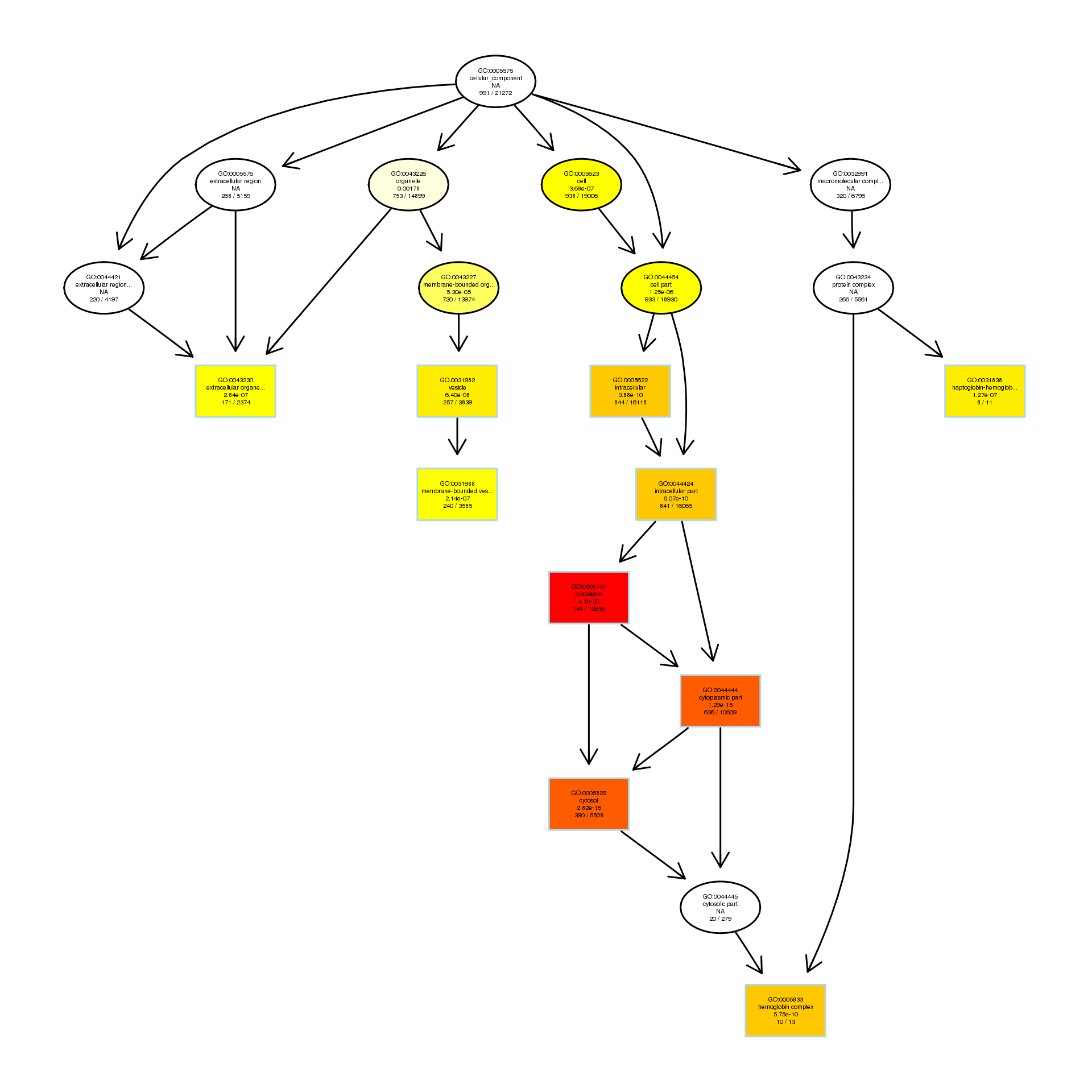

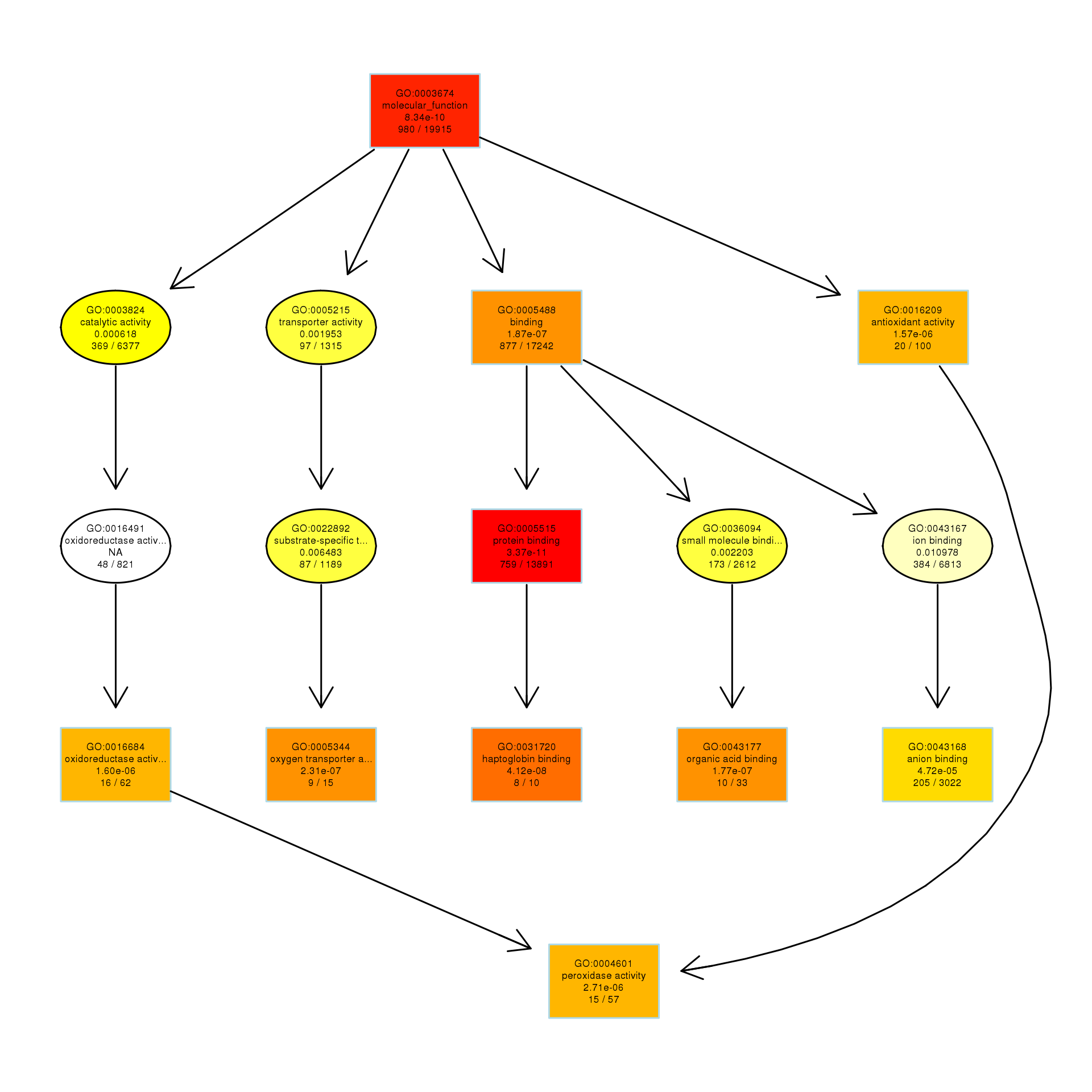


(b) (c)
